# Pseudogout-Induced Cervical Myelopathy: A Report of Two Cases and Review of the Literature

**DOI:** 10.7759/cureus.85145

**Published:** 2025-05-31

**Authors:** Mohamed A. A Ibrahim, Mohamed Negm, Mohamed G Abdelkader, Mohamed F Elhalawany, Lotfy M Shwitter

**Affiliations:** 1 Orthopedic Surgery, Al-Azhar University, Cairo, EGY

**Keywords:** calcium pyrophosphate dihydrate deposition, cervical myelopathy, posterior spinal fusion, pseudogout, spine

## Abstract

Calcium pyrophosphate dihydrate deposition (CPPD) disease, also known as pseudogout, is the most common cause of calcification of ligamentum flavum (CLF) compared to other degenerative conditions. Symptomatic CLF is a rare cervical spine disorder that leads to spinal cord compression, resulting in myelopathic symptoms. We report two rare cases of pseudogout-induced cervical myelopathy, along with a review of the literature. Both patients were seen at Al-Azhar University Hospital in Cairo, Egypt, presenting with severe neck pain, gait disturbances, and hand clumsiness. The first patient had these symptoms for two months, while the second patient experienced them for three months. The second patient also had restricted motion in extension and rotation, as well as shooting pain in both upper extremities. Cervical spine MRI revealed posterior cord compression and myelomalacia at the C3-C6 levels in the first patient and at the C4-C5 levels in the second patient. Pseudogout (CPPD) disease was suspected as the cause of spinal cord compression, and histopathological analysis of the deposits found at the site of compression during the decompression procedure confirmed the diagnosis. The deposits were characterized by rhomboid blue calcium crystals that were mildly birefringent, distinguishing them from the needle-shaped crystals seen in gout. At the four-week postoperative follow-up, both patients showed significant improvement in clinical and functional outcomes, as measured by the Japanese Orthopaedic Association score. In summary, CLF due to pseudogout (CPPD deposition) is a rare but clinically significant cause of cervical myelopathy. Histopathological examination is crucial for a definitive diagnosis. Early posterior cervical decompression with instrumentation appears to be an effective treatment, though larger studies and long-term follow-up are necessary to confirm these findings and optimize management strategies.

## Introduction

Pseudogout, or calcium pyrophosphate dihydrate deposition (CPPD) disease, is an inflammatory arthropathy that most commonly affects the knee and elbow. Spinal pseudogout, which is rare, typically presents with neck discomfort as the primary clinical sign. CPPD deposition in the ligamentum flavum of the cervical spine is extremely rare, with only a few reports in the English-language literature, primarily from Japan, most of which are case reports and reviews of collected cases, emphasizing the rarity of this condition [1,2-4].

Pseudogout is characterized by the accumulation of CPPD crystals in articular and periarticular tissues [5]. There are few instances of cervical myelopathy caused by subaxial CPPD deposition, and CPPD deposition in the cervical spine has been described in the literature. Crowned dens syndrome (CDS), also known as retro-odontoid CPPD deposition, typically presents with neck stiffness and pain, which can progress to myelopathy and spinal cord compression [6].

Due to the gradually progressive nature of CPPD and the resulting cervical myelopathy caused by calcification of ligamentum flavum (CLF), early identification and treatment are essential to improving the patient's overall quality of life [3].

When a posteriorly positioned lesion is present, the differential diagnosis for compressive cervical myelopathy includes pseudogout, gout, ossification of the ligamentum flavum (OLF), infection, and malignancy [7].

OLF in the cervical spine is less common than CPPD deposition in the cervical ligamentum flavum. It is widely accepted that these two conditions are histologically distinct. OLF typically affects a single level, and there is no common site in the cervical spine. Differences in MRI findings, such as the side of the involved ligament, lack of continuity with the lamina, acute or chronic features (e.g., MRI peri-nodular edema in CPPD cases), the presence of a retro-odontoid mass, and MRI morphology (nodular in CPPD versus mound-like in OLF), further distinguish CPPD deposition from OLF [2].

Posterior decompression creates additional space for the spinal cord and allows for direct removal of the compressive lesion. Cervical spine decompression and fusion is the most common treatment for multilevel cervical spondylosis with myelopathy, with or without radiculopathy [4,8]. Including posterior cervical fusion helps prevent post-laminectomy instability and subsequent kyphosis [8].

Since Roy-Camille et al. first proposed posterior cervical fixation with lateral mass screws in 1979, this technique has become increasingly common for treating various cervical spine conditions [9].

## Case presentation

Case 1

A 64-year-old diabetic female presented with a two-month history of neck pain and stiffness, gait difficulty, clumsy hands, and preserved sphincteric function.

**Figure 1:**
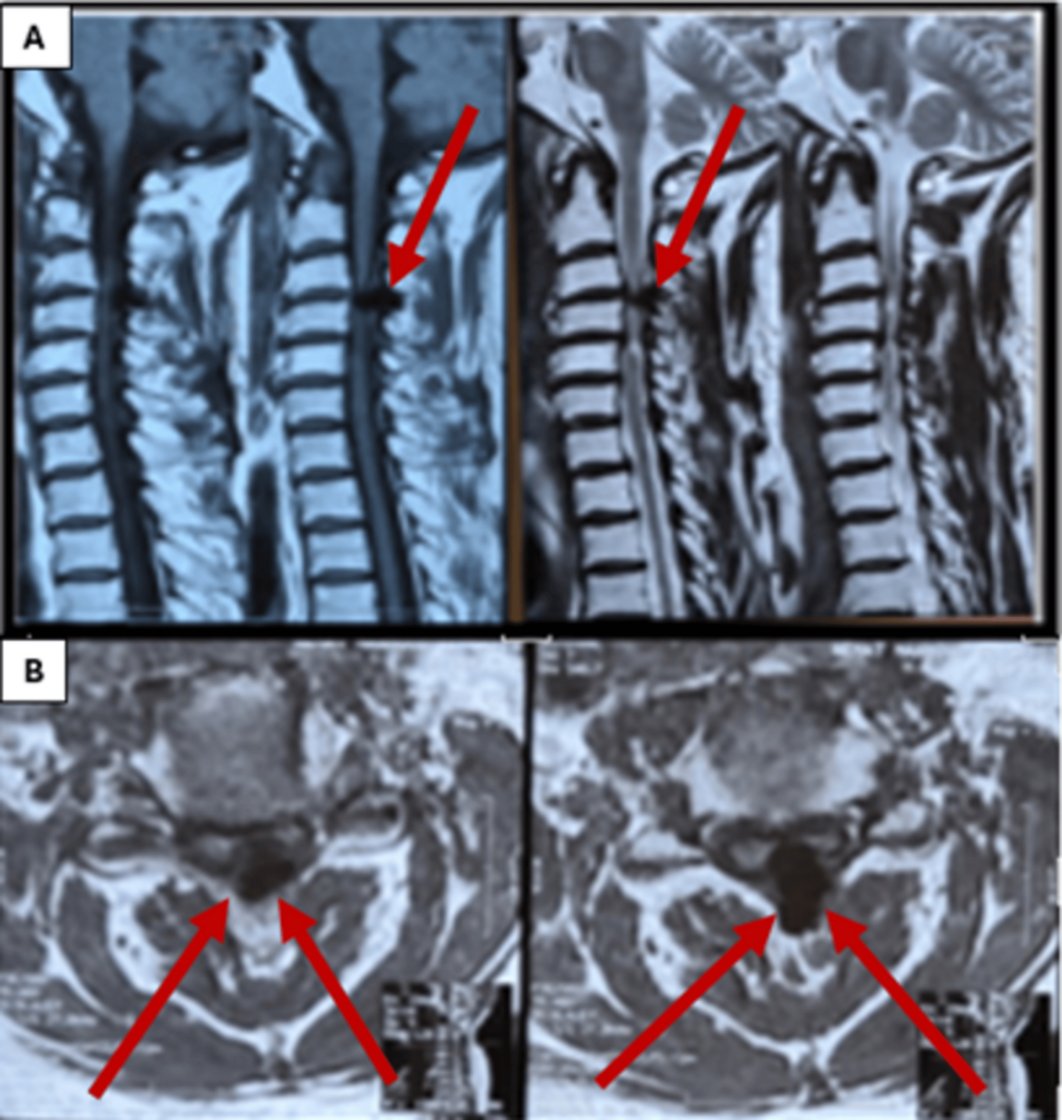
Preoperative cervical spine MRI (A) Sagittal view and (B) axial view showing posterior cord compression by a nodular lesion in the ligamentum flavum at the C3-C4 and C4-C5 levels. The low signal intensity on both T1- and T2-weighted images indicates a calcified lesion. Additionally, intramedullary high signal intensity (myelomalacia) is present at the affected levels.

Physical examination revealed limited cervical spine movement, hypertonia, hyperreflexia, a positive Babinski sign, ankle clonus, a positive grip and release test, a positive inverted radial reflex, and a positive Hoffmann reflex. Cervical spine MRI showed posterior cervical cord compression and myelomalacia at the C3-C6 level. The preoperative Japanese Orthopaedic Association (JOA) score was 12 (Figure 1A, 1B). 

**Figure 2:**
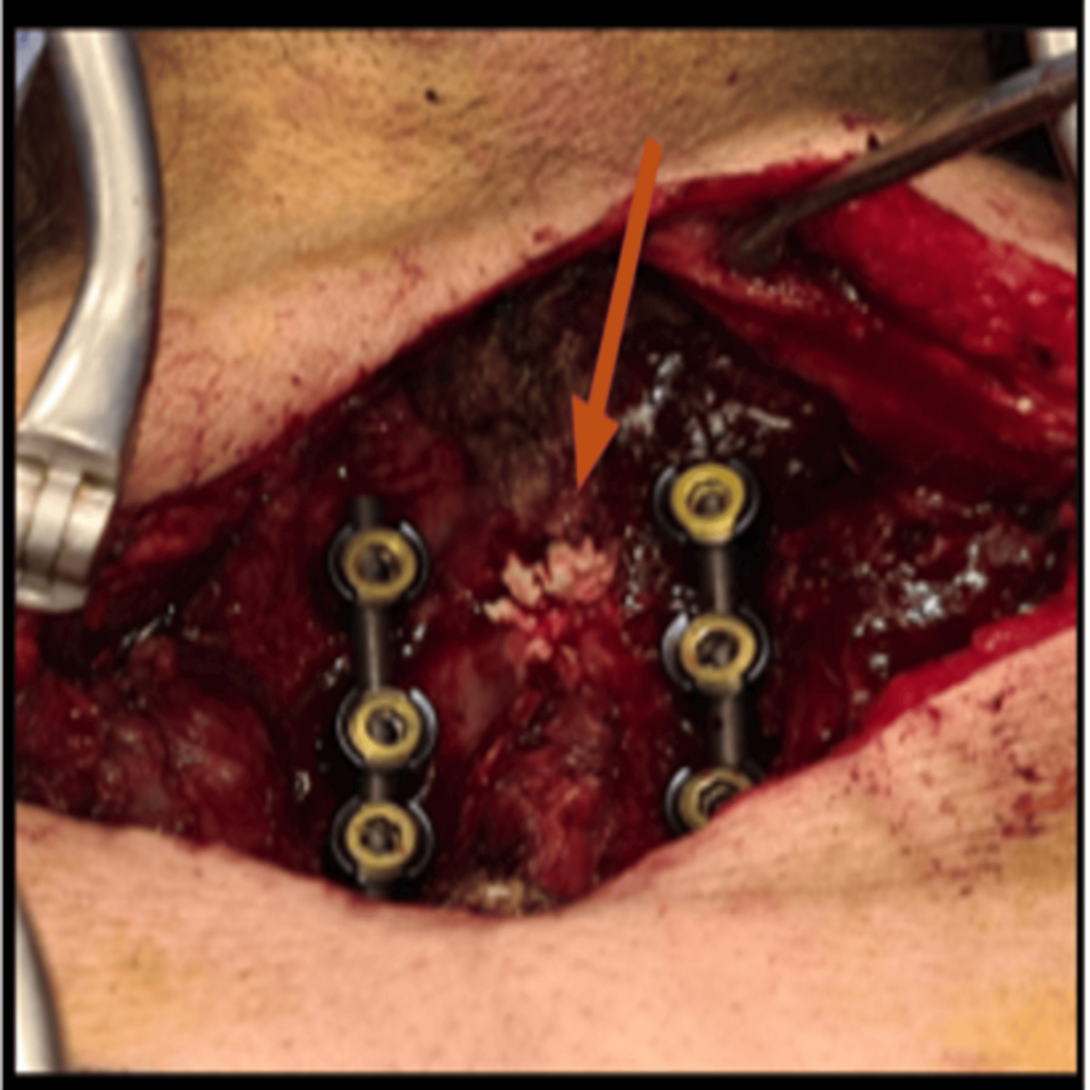
Intraoperative images showing calcified deposits in the ligamentum flavum compressing the spinal cord posteriorly at the C3-C6 levels

**Figure 3:**
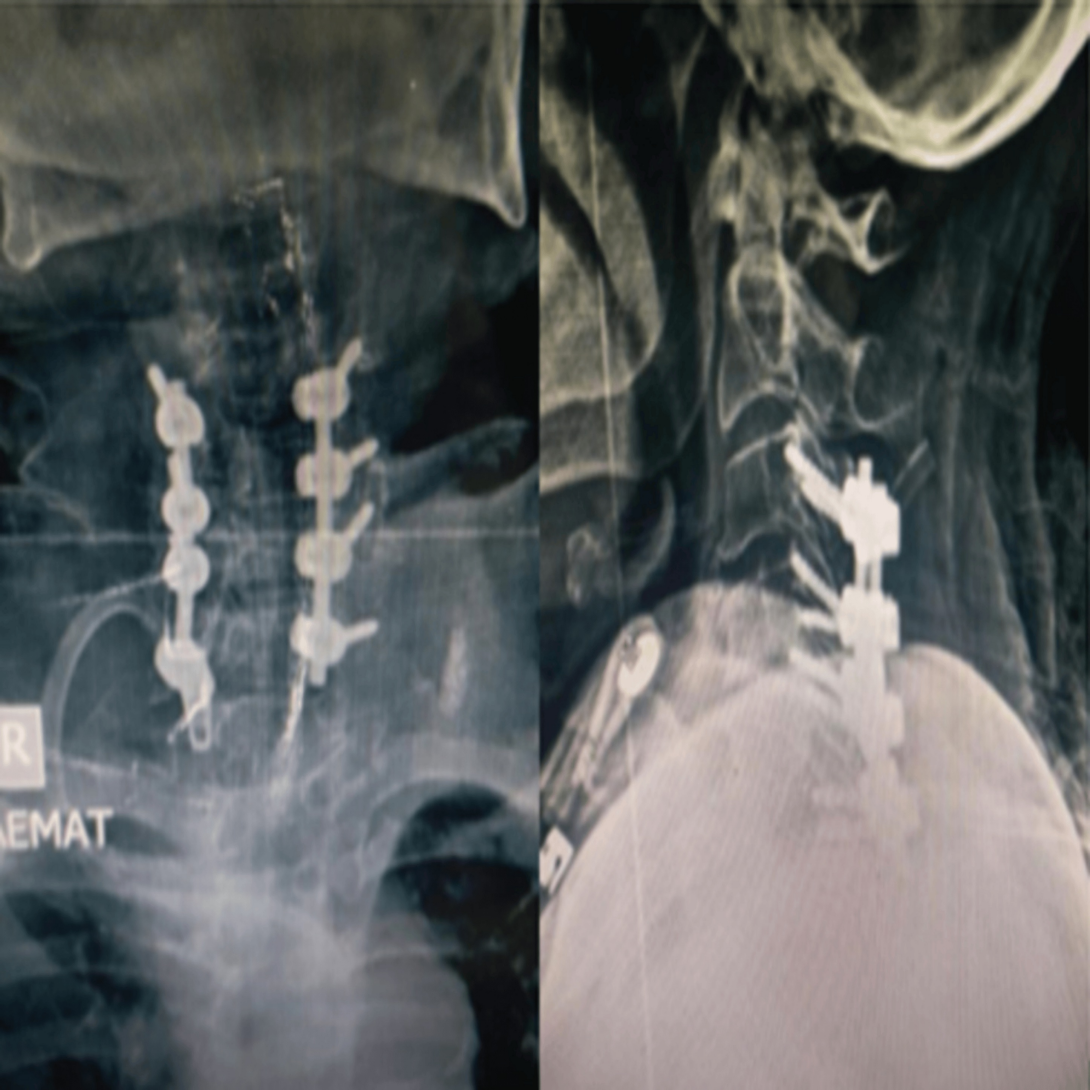
Postoperative plain X-ray of the cervical spine (A) Anteroposterior (AP) view and (B) lateral view showing C3-C6 decompression and lateral mass fixation.

The patient underwent posterior cervical decompression and lateral mass fixation at C3-C6, with a sample taken from the deposits within the ligamentum flavum for histopathological examination to confirm the diagnosis. Her symptoms improved after surgery. The postoperative JOA score at four weeks was 15, with a calculated recovery rate of 60%. At the last follow-up, three months postoperatively, the functional outcome and recovery rate remained stable, with no neurologic deterioration (Figure 2, Figure 3).

Case 2

A 58-year-old hypertensive man presented with a three-month history of severe neck pain and shooting pain in both upper extremities.

**Figure 4:**
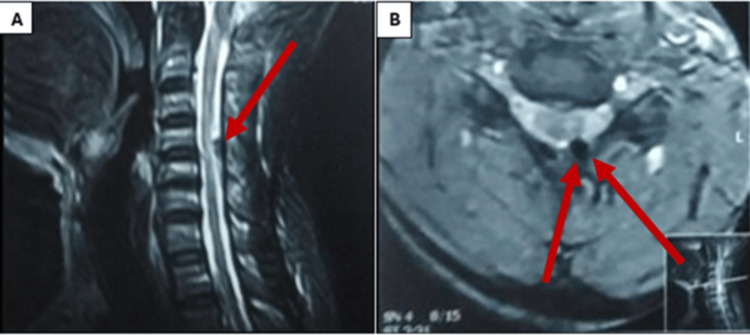
Preoperative cervical spine MRI (A) Sagittal view and (B) axial view showing posterior cord compression by a nodular lesion in the ligamentum flavum at the C4-C5 level. The low signal intensity on both T1- and T2-weighted images indicates a calcified lesion. Additionally, intramedullary high signal intensity (myelomalacia) is observed at the affected level.

His physical examination showed limited motion in extension and rotation, hand clumsiness, unsteady gait, hypertonia and hyperreflexia, positive Babinski and ankle clonus, a positive grip and release test, and preserved sphincteric function. Cervical spine MRI demonstrated posterior cervical cord compression and myelomalacia at the level of C4-C5. The preoperative JOA score was 10 (Figure 4).

**Figure 5:**
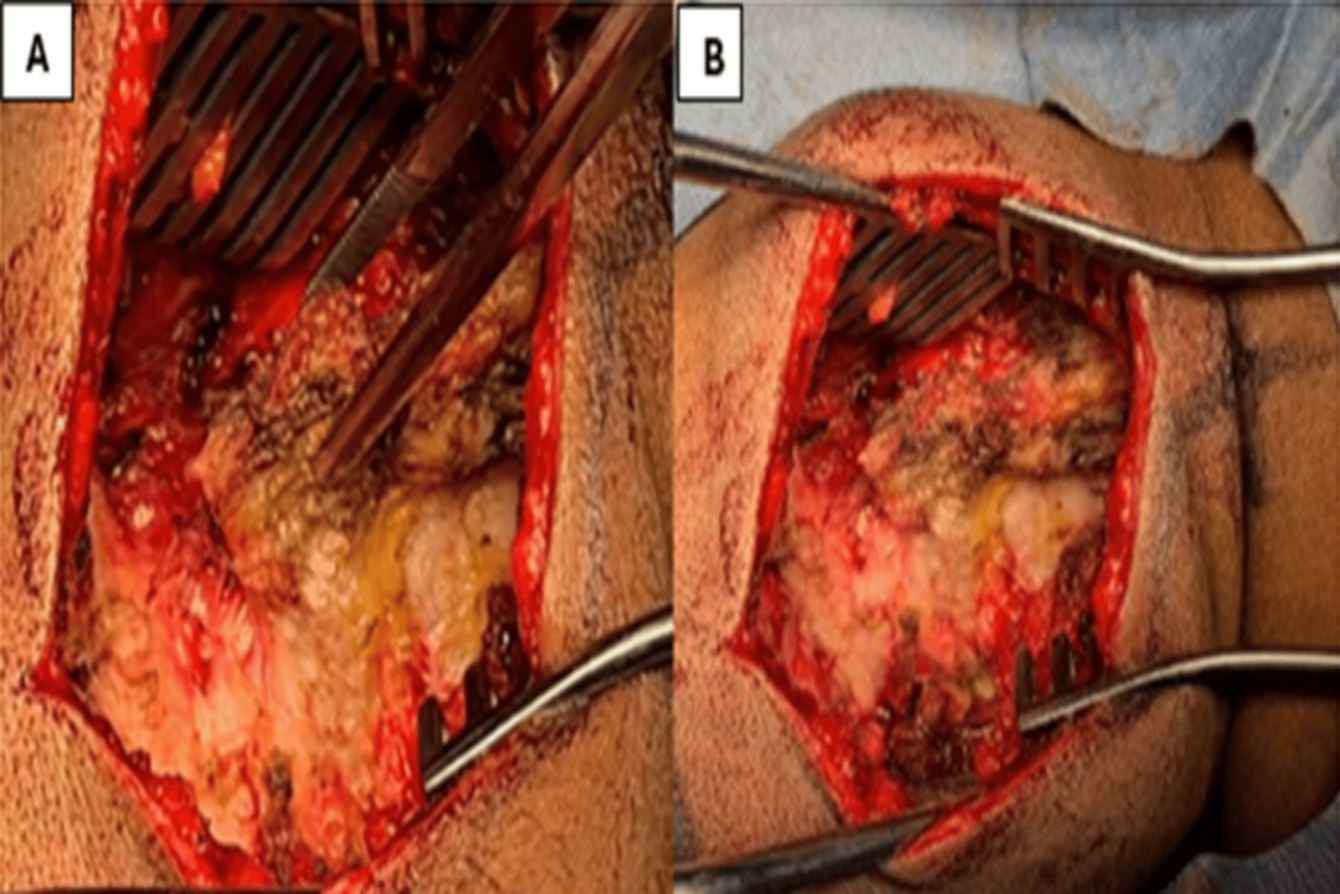
Intraoperative images showing calcified deposits in the ligamentum flavum (A) and (B) compressing the spinal cord posteriorly at the C4-C5 level

**Figure 6:**
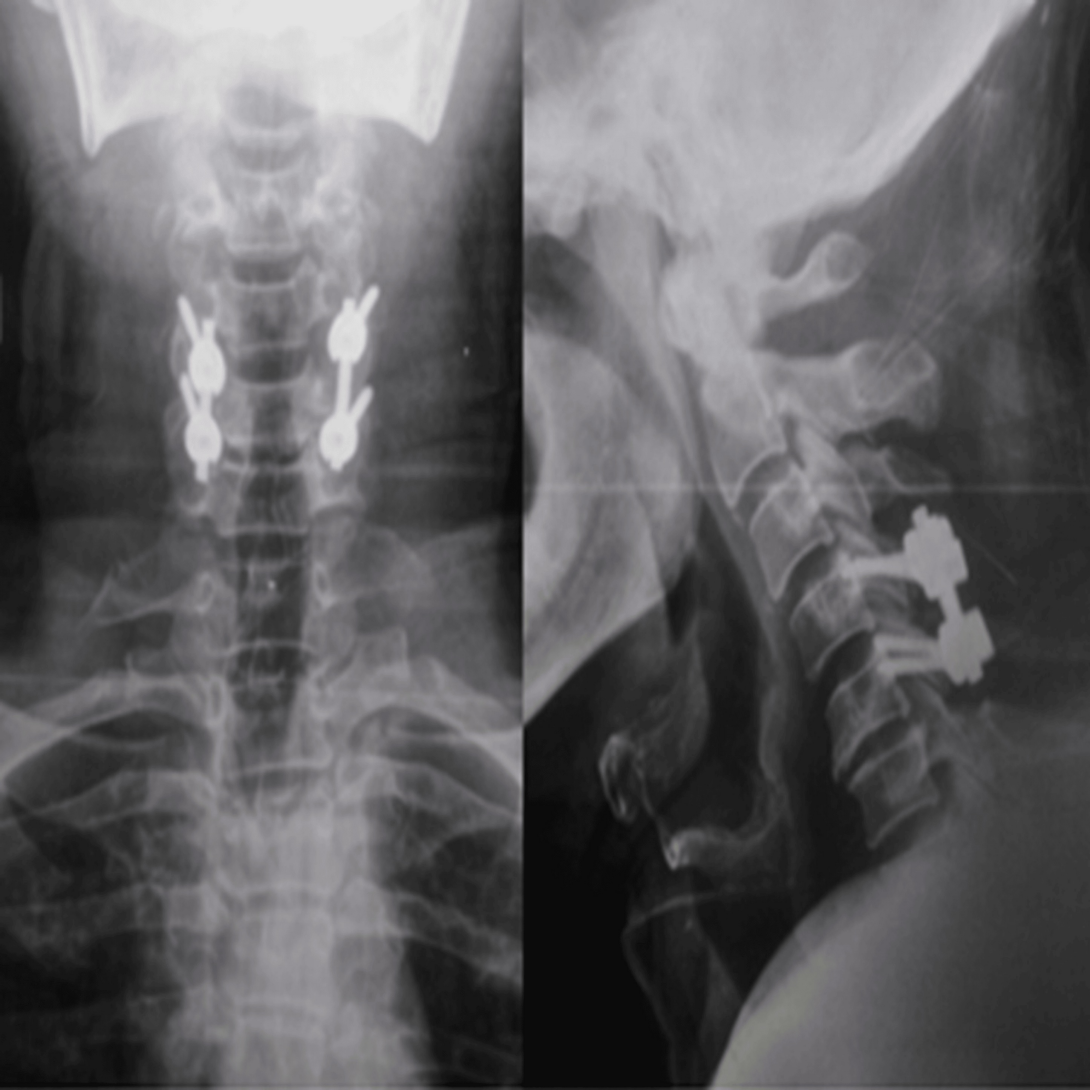
Postoperative plain X-ray of the cervical spine (A) AP view and (B) lateral view showing C4-C5 decompression and lateral mass fixation.

The patient also underwent posterior cervical decompression and lateral mass fixation at C4-C5, with samples taken from the deposits noted in the ligamentum flavum and facet joints for histopathological examination to confirm the diagnosis. His symptoms improved after surgery. The postoperative JOA score at four weeks was 14, with a calculated recovery rate of 57%, which remained unchanged at the last follow-up three months after the operation (Figure 5, Figure 6).

Regarding the improvement of clinical signs and symptoms in both cases after the operation, pain and sensory disturbances in the upper limbs, along with clumsy hands, were the first to show improvement. Gait difficulty also showed some progress, while spasticity continued to improve gradually over time. Reevaluation using the JOA score at six and 12 months postoperatively is planned to monitor for any neurological deterioration or further improvement.

The demographic, radiographic, and clinical data of the two case reports are presented in Table 1.

**Table 1:** Demographic, radiographic, and clinical data of the two case reports JOA, Japanese Orthopaedic Association

Parameter	Case 1	Case 2
Age	64 years	58 years
Sex	Female	Male
Imaging findings	Posterior cervical cord compression and myelomalacia at C3-C6	Posterior cervical cord compression and myelomalacia at C4-C5
Neurological manifestations	Clumsy hands, gait difficulty, hypertonia, and hyperreflexia	Clumsy hands, unsteady gait
Surgical levels	Posterior cervical decompression and instrumented fusion at C3-C6	Posterior cervical decompression and instrumented fusion at C4-C5
Preoperative JOA score	12	10
Postoperative JOA score (four weeks and three months)	15	14

Histopathology examination

**Figure 7:**
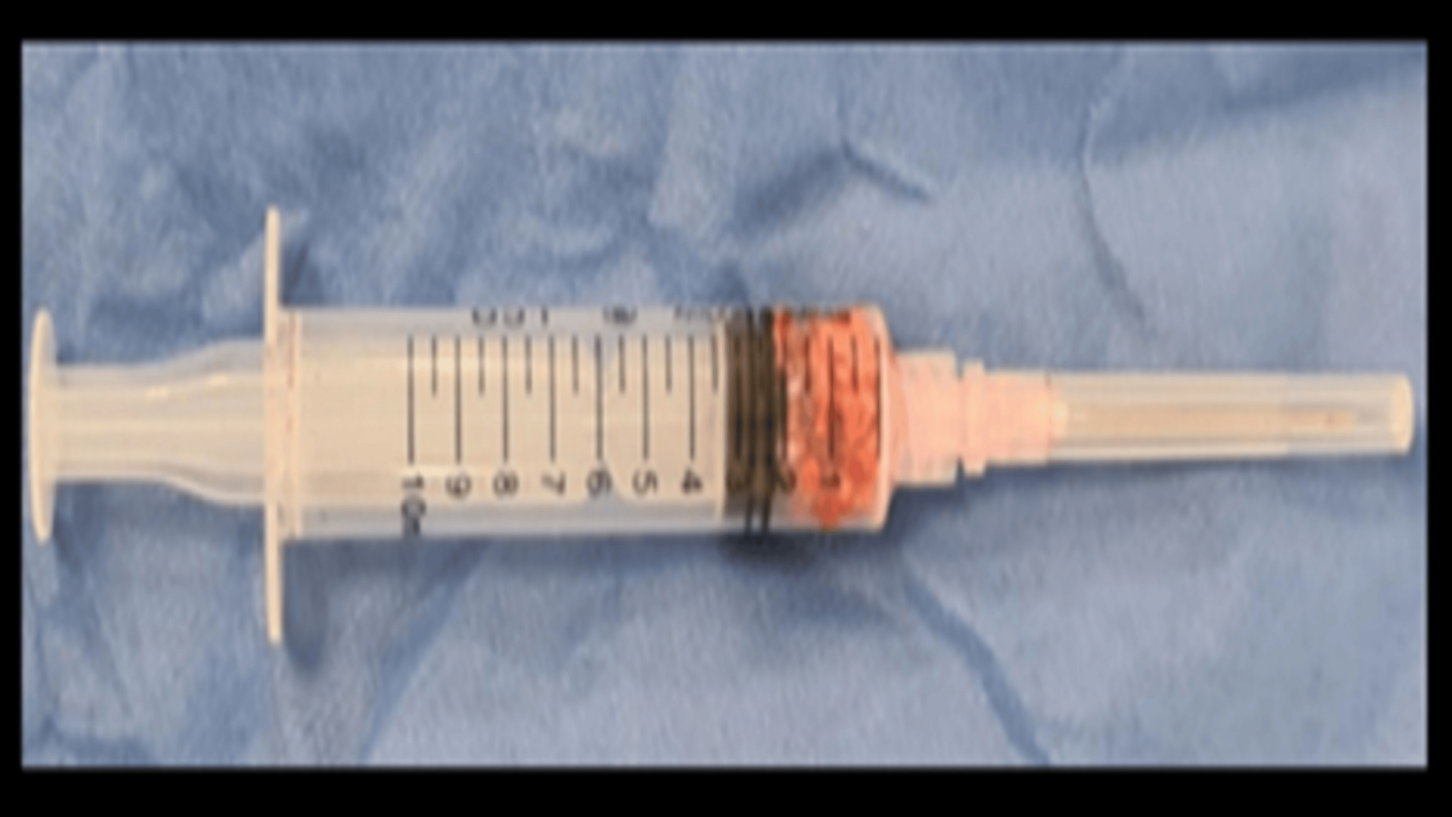
Calcium pyrophosphate crystal deposits sample for histopathological examination

An excisional biopsy was taken intraoperatively from the deposits noted in the ligamentum flavum at the site of compression. Histopathological analysis confirmed the definitive diagnosis of CPPD disease, revealing multiple irregular fibrofatty muscular tissue fragments containing calcium pyrophosphate crystal deposits (Figure 7).

Microscopic examination using polarized light microscopy and H&E staining revealed fragments of fibrocartilage and ligamentum flavum with extensive deposition of birefringent rhomboid-shaped blue calcium pyrophosphate crystals. No evidence of granulomatous inflammation or malignant tissue was observed. 

## Discussion

Pseudogout is an inflammatory arthropathy caused by the accumulation of CPPD crystals, which are associated with dysfunctions in various phosphatases and transporters [10]. CPPD is uncommon in individuals under 50 years of age, with its prevalence increasing significantly with age [6]. Risk factors for CPPD disease include loop diuretics, osteoarthritis, and hyperparathyroidism [11]. Since phosphate crystal deposition can occur in articular cartilage, tendons, and ligaments, CPPD is typically regarded as a disease of peripheral joints [12]. Although rare, spinal pseudogout has been reported to affect structures such as ligaments, articular cartilage, synovial joints, or intervertebral discs [6]. Most reported cases of spinal pseudogout involve the transverse ligament of the atlas, potentially leading to CDS [13]. Almadhoun and Ta'amneh emphasize the importance of considering CPPD in the differential diagnosis of acute neck pain, especially when myelopathy is present [4]. As in our cases, spinal cord compression in the cervical spine due to pseudogout involving the ligamentum flavum has been rarely described in the literature [6].

The clinical manifestations of cervical spine pseudogout depend on the extent of neural structure involvement. Sekijima et al. reported that the predominant symptoms include fever, stiffness, and acute posterior neck pain [14]. When calcified ligaments cause significant compression of the spinal cord or nerve roots, patients may develop myelopathy, myeloradiculopathy, or radiculopathy [6].

To confirm the diagnosis of cervical compressive myelopathy due to CPPD deposition in the ligamentum flavum, a thorough correlation of clinical findings with imaging studies is essential. Histopathological examination remains a crucial diagnostic tool, as clinical symptoms and radiologic findings do not always align [9].

According to Turaga et al. [6], several disorders may present similarly, including cervical myelopathy caused by CLF from CPPD deposits or CLF with other crystal types. Haikal et al. [15] noted some CLF cases with no evidence of CPPD deposits. Similar cases of cervical myelopathy due to CPPD deposition in the ligamentum flavum have been described in the literature. These typically involve elderly female patients presenting with neck pain and symptoms of cervical myelopathy. In most cases, the mid to lower cervical spine is affected, and surgical intervention is often required, similar to the cases we present here. While the precise etiology of CPPD deposition disease remains unclear, it has been linked to various metabolic and endocrine conditions, including diabetes mellitus, hypertension, hyperparathyroidism, hypophosphatasia, hemochromatosis, gout, and rheumatoid arthritis [16,17].

As described by Haikal et al. [15] and ELNemr et al. [16], our first case involved a 64-year-old female patient with diabetes, and the second case involved a 58-year-old hypertensive male patient. Both presented with neck pain, gait disturbances, and clumsiness of the hands. In both cases, MRI confirmed posterior cord compression and myelomalacia caused by a calcified lesion, extending from C3 to C6 in the first case and localized at C4-C5 in the second.

Oshima et al. [18] noted that the JOA score, which ranges from 0 to 17, is widely used by orthopedic spine surgeons in Japan to assess the severity of cervical myelopathy. The score evaluates neurological function across six domains: motor function of the upper and lower extremities, sensory function of the upper extremities, trunk, and lower extremities, and bladder function. It is also used to monitor recovery after conservative or surgical treatment.

In our cases, the JOA score was recorded preoperatively, at four weeks postoperatively, and again at three months postoperatively. Follow-up assessments were scheduled between six to twelve months. The recovery rate was calculated using the Hirabayashi method: postoperative JOA − preoperative JOA / 17 − preoperative JOA × 100. Once a diagnosis is suspected, decompression surgery is the most effective intervention for cord compression caused by a calcified cervical ligamentum flavum. In a study by Moon et al. [19], many patients showed favorable outcomes following a standard laminectomy procedure, which involves the removal of the calcified mass. If the crystals extend to the cervical facet joints, posterior fusion is necessary to maintain spinal stability. According to ELNemr et al. [16], most patients experienced uneventful postoperative recoveries, with significant improvement in their myelopathic symptoms.

## Conclusions

CLF due to pseudogout (CPPD deposition) is a rare but clinically important cause of cervical myelopathy. Histopathological examination remains crucial for establishing a definitive diagnosis. Early posterior cervical decompression with instrumentation has been shown to be an effective treatment approach in these cases. However, larger studies with long-term follow-up are necessary to validate these findings and guide optimal management strategies.
